# LEXF-ATT-XLM: A hybrid lexicon-enhanced attention model for hate speech detection in low-resource language Roman Urdu

**DOI:** 10.1371/journal.pone.0354875

**Published:** 2026-08-20

**Authors:** Jaweria Jalil Awan, Muhammad Hamid, Tagrid Abdullah N. Alshalali

**Affiliations:** 1 Department of Computer Sciences, Government College Women University Sialkot, Sialkot, Pakistan; 2 Department of Information Systems, College of Computer and Information Sciences, Princess Nourah bint Abdulrahman University, Riyadh, Saudi Arabia; Khalifa University, UNITED ARAB EMIRATES

## Abstract

Hate speech detection in low-resource and informally written languages remains a significant challenge due to the lack of annotated corpora, orthographic variability, and complex code-mixing. Roman Urdu a non-standardized variant of Urdu written in the Latin script exemplifies these linguistic hurdles. In this paper, we propose LEXF-ATT-XLM, a novel hybrid deep learning architecture that synergizes contextual language modeling with explicit domain knowledge. Our model leverages XLM-RoBERTa for deep contextual embeddings, passed through a two-layer Bidirectional Gated Recurrent Unit (BiGRU) and a multi-head attention mechanism to capture both sequential and salient linguistic patterns. Crucially, the pooled representations are fused via a learnable gating layer (Linear + tanh) before final classification. Furthermore, we integrate a domain specific Roman Urdu hate lexicon as an auxiliary regression supervision signal within a multi task learning framework to guide the model’s focus. Evaluated on the RU-HSD-30K dataset using 3-fold stratified cross-validation, the proposed model achieves an average accuracy of 88.83% and a weighted F1 score of 88.83%, with fold wise weighted F1-scores of 88.58%, 89.19%, and 88.71%. Extensive ablation studies confirm that the lexicon-guided auxiliary supervision significantly enhances the model’s ability to handle lexical variations, negations, and informal spelling. These findings demonstrate the robust effectiveness of our approach in addressing the unique linguistic challenges of Roman Urdu hate speech detection.

## 1. Introduction

The spread of toxic, abusive, and hateful materials on social media has become a serious challenge to online safety and user wellness, making automated hate speech detection an important task within the framework of Natural Language Processing (NLP). Although there are strong solutions to standardized and high resource languages, such as English, progress in low resource and informally written languages is limited. An example is Roman Urdu a non-standardized form of Urdu in the Latin script widely used throughout South Asia and by the global diaspora on social media platforms like Facebook, Twitter and YouTube [1,2].

Detection of Hate speech in the Roman Urdu language poses a special linguistic challenge. The language has no standard orthography, has a high lexical and morphological variability, and is often code-mixed with English [1,3]. An example of this is where one concept is represented by several orthographical variants (e.g., “nai acha,” “nai achaaa,” “na achha”), thus seriously constraining the effectiveness of conventional tokenization and classification methods. Moreover, hate speech here is highly reliant on sociolinguistic overtones, sarcasm, and implicit slurs, which are incredibly hard to detect. The works by Azam et al. [1] and Bilal et al. [2] were the first to utilize augmented datasets (RUSOLD, RUT, and RU-HSD-30K) and attention-based Bi-LSTM models. More recent models, including BERT-RU [4] and Passion-Net [5], have shown that a combination of transformer architectures with deep learning models can result in major advancements. Moreover, Malik et al. [6] as well as others [7,8] investigated multilingual versions and paradigms that integrate MBERT with traditional classifiers (SVM, Random Forest) to increase the accuracy.

Even with these developments, there remains a huge gap in research. Most current models consider contextual and lexical signals as separate entities, usually not considering sequence-level contingencies that are critical to effective culture-dependent hate detection [9–11]. Moreover, existing hybrid architectures generally use basic feature concatenation, which can frequently cause “feature dilution” where the low density but vital lexical signal is overwhelmed by high-dimensional deep representations [12–14]. Moreover, the specific features of linguistics (hate lexicon intensity) are seldom directly incorporated into the learning objective and do not provide interpretability and sensitivity to nuanced hate speech.

The study propose the LEXF-ATT-XLM model in order to address these gaps which defines a structured, learnable form of interaction between deep contextual embeddings and explicit domain-specific lexical cues. The major contributions of this work include:

1. The study proposed a new hybrid model that combines multilingual contextual embeddings (XLM-RoBERTa), sequential modeling (BiGRU) as well as multi-head attention. In contrast to the previous models, we make use of a learnable gating layer (Linear + tanh) to combine these representations which are effective in capturing the overall semantic context and localized hate-specific dependencies.
2. We use a strong multi-task learning goal in which domain-related hate lexicon scores are used to act as an auxiliary regression signal. This method regularizes the network and forcing the model to be consistent with lexical hate patterns without the feature dilution which is caused by mere concatenation.
3. We design a special preprocessing approach which is effective in reducing the noise of Roman Urdu. Through a negation- prefix transformation, we maintain sentiment polarity, and ensure that the transformer encoder differentiates between negated and affirmed lexical units, and hence this greatly reduces ambiguity in polarity.
4. With cross-validation on the RU-HSD-30K dataset using a 3-fold stratified cross-validation, we are able to show that our multi-task framework achieves a weighted F1-score of 88.83%. Our ablation experiments validate this claim as every component has a cumulative effect, meaning that lexicon-directed auxiliary supervision yields the largest performance improvement in detecting subtle and implicit hate.

The rest of the paper is organized as follows: Section 2 is a review of the related studies on hate speech detection and the application of large language models in low-resource settings. Section 3 is a detailed description of the methodology. Section 4 is a discussion on the experimental results. Section 5 is a summary of the concluding remarks and the future research directions.

## 2. Related work

Detection of hate speech has been recognized as an essential NLP task. This task has also received significant attention due to its relevance to multilingual social media platforms. However, difficulties such as lack of standardization and morphological complexities make hate speech detection a complex NLP task. This section presents an overview of the recent developments in four key areas: dataset development, classical and deep learning models, transformer models, and cross-domain approaches.

### 2.1. Dataset development and preprocessing in Roman Urdu

Research has also been conducted to augment Roman Urdu hate speech datasets to overcome the scarcity and noise of the data. Azam et al. [1] used generative augmentation with MT5 and mBERT-MLM models to augment the RUSOLD and RUT datasets, achieving a maximum of 86% F1-score. Bilal et al. [2] introduced the RU-HSD-30K dataset, containing 30,000 posts, with normalization and phonetic mapping using UrduPhone, and achieved a maximum of 88.5% F1-score using the Bi-LSTM model with attention. Other datasets include RU-PHS [7] with 5,002 geo-tagged tweets, and BERT-RU [4], pretrained on 173K messages, achieving 97.25% F1. Other related work, such as that of Atif et al. in [3], further added more fine-grained labeling for hate types, while PURUTT in [15] and ORUD-Detect [16] provided larger corpora, using hybrid models or fine-tuned LLMs to surpass 90% F1.

### 2.2. Classical and deep learning approaches

Earlier methods used SVMs, Random Forests, and TF-IDF features but were not effective in handling out-of-vocabulary words and their ambiguity. GRUs with attention have also been used to achieve competitive F1-scores on smaller datasets in research papers like [17]. Hybrid models such as Passion-Net [5] and MBERT-SVM pipelines have also been used to achieve better results than transformer-based models on Roman Urdu datasets. BERT-based models have also been used to achieve good results, i.e., 94% F1-score.

### 2.3. Multilingual and transformer-based architectures

The performance of multilingual transformers is impressive in low-resource hate speech detection. XLM-RoBERTa and MuRIL, fine-tuned models, have shown more than 90% accuracy in Urdu and Indic hate speech detection tasks [6,18]. RomanBERT, a model that uses fastText subword-level embeddings, obtained an F1-score of 0.95 in the Roman Urdu hate speech detection task, which proves the effectiveness of semantic representation. QLoRA allows memory-efficient fine-tuning of large language models using low-rank adapters and quantization, which retains the performance of models in low-resource settings [19]. This is a low-rank adaptation of multilingual transformers, which has shown effectiveness in hate speech detection. In addition, lightweight transformer models, such as DistilBERT, have shown impressive performance with lower complexity [20]. This proves the effectiveness of efficient transformer models. Interpretability techniques, such as LIME, are used to increase transparency by explaining category-level predictions [21].

### 2.4. Cross-domain, multimodal and informal contexts

Studies on code-mixing, cross-domain, and multimodal settings emphasize the significance of preprocessing. Bi-LSTM with EfficientNet [22], ELMo and LASER embeddings [23], and CMSA-mBERT on code-mixed Roman Urdu–Punjabi text [24] emphasize that the model’s performance is largely dependent on the preprocessing phase. Other associated tasks such as sarcasm detection [25], insult detection [26], and hate speech/offensive text detection [27] emphasize the significance of the joint effect of lexical and contextual features.

### 2.5. Gaps and motivation for our approach

While prior studies show promising results, they reveal key gaps:

- Most models treat contextual and lexical signals independently, limiting nuanced understanding.
- Lexicon-based cues such as slurs and profanities are underutilized in transformer pipelines.
- Negation handling and fine-grained hate intensity modeling are often absent.
- Few works incorporate auxiliary supervision or explicitly guide models using hate lexicon gradients.

To address these gaps, we propose LEXF-ATT-XLM, a hybrid model that fuses multilingual contextual embeddings (XLM-R), sequential structure (BiGRU), and a novel lexicon-aware auxiliary objective. Our model incorporates hate lexicon intensity scores using an auxiliary supervision objective in a multi-task learning setup. Domain-specific preprocessing especially negation-aware normalization further enhances our model’s robustness to Roman Urdu’s noisy and unstructured nature. As illustrated in Table 1 Comparative Analysis of Existing Studies on Roman Urdu Hate Speech Detection (2022–2025), our approach significantly outperforms prior models by effectively addressing the linguistic challenges unique to Roman Urdu through a multi-pronged architectural and preprocessing strategy.

**Table 1:** Summary of related studies on Roman Urdu hate speech detection, including datasets, models, and key results.

Ref	Year	Title (Abbreviated)	Dataset	Labels	Best Model	Results (Acc/F1)	Key Advantages	Limitations
1	2022	Data Augmentation for R. Urdu	RUSHOLD, RUT	Multi-label	MT5 + CNN-gram	76% F1 (RUSHOLD), 86.48% F1 (RUT)	Reduces annotation cost	Modest F1 gains
2	2022	Context-Aware Bi-LSTM	RU-HSD-30K	Hate/Neutral	Bi-LSTM + Attention	87.5% Acc, 88.5% F1	Custom normalization	No sarcasm handling
3	2023	Geo-Spatial Mapping	RU-PHS (5K tweets)	Political Offensive/Neutral	FFNN + fastText	93% Acc/F1	Spatial analysis	Small dataset
4	2023	Transformer (BERT-RU)	RU-HSD-30K + 173K texts	Hate/Neutral	BERT-RU	96.7% Acc, 97.25% F1	First R. Urdu BERT	Underperforms vs. BERT-English
5	2024	Attention (Passion-Net)	RUHSOLD	Coarse/Fine-grained	RU-ULMFIT + Dual Attention	98.7% Acc (Coarse), 92.6% (Fine)	SOTA, explainable	Fine-grained class confusion
6	2024	Hybrid ML-DL	HS-RU-20, RUHSOLD	Multi-class	CNN/LSTM + MBERT	0.95 F1 (Coarse)	Auto feature extraction	Model complexity
7	2024	BERT for R. Urdu	30K tweets	Hate/Non-Hate	BERT	93.5% Acc, 94% F1	Robust for low-resource	Needs normalization
8	2024	Hybrid ML Models	HS-RU-20, RUHSOLD	Multi-class	SVM + MBERT	0.93-0.95 F1	Combines DL + ML	Non-standard language
9	2023	Multilingual XLM-R	3,713 tweets (Eng/Urdu)	Threatening/Non-threatening	XLM-RoBERTa	92% Acc, 90% F1	Handles low-resource	Small dataset
10	2023	UHated (RoBERTa)	7,871 Urdu tweets	Hate/Offensive/Neutral	RoBERTa	85% Acc, 82% F1	Handcrafted lexicon	Low IAA (42.9%)
11	2023	Urdu-BERT	2,400 Urdu tweets	Threatening/Target	Urdu-BERT	87.5% Acc, 87.8% F1	Contextual understanding	Computational cost
12	2023	Multilingual (IndicBERT)	Assamese/Bengali/Bodo	HOF/NOT	IndicBERT	69-83% F1	Tailored per language	Overfitting
13	2024	Cyberbullying (GRU)	RU Dataset	Multi-class	GRU	97% Acc/F1	User profiling	No emoji analysis
14	2024	Multilingual FastText	13 datasets	Hate/Non-Hate	SVM + RomanBERT	95% F1	Subword embeddings	Spelling variations
15	2024	XLM-RoBERTa + LIME	English/Urdu/Sindhi	Fine-grained	XLM-RoBERTa	91% F1 (Binary)	Explainable AI	Low Urdu performance
16	2024	Code-Mixed XLNet	170K+ instances	Hate/Neutral	XLNet	96% Acc	Handles code-mixing	Binary-only
17	2025	Multimodal (BiLSTM)	MMHS11K	Hate/No-Hate	BiLSTM + EfficientNetB1	81.5% Acc (Urdu)	Text + image context	OCR limitations
18	2025	LASER Embeddings	11 languages	Hate/Non-hate	LASER + LR	High F1 (low-resource)	Multilingual support	Contextual nuances
19	2025	PURUTT Corpus	72K parallel texts	Toxic/Non-toxic	Ensemble (33 models)	97.07% Acc	Large-scale corpus	Class imbalance
20	2025	ORUD-Detect (SVM/Bi-LSTM)	89K comment	Offensive/Non-offensive	Bi-LSTM	98% F1	Handles informal text	Computationally heavy
21	2021	CNN for Urdu/R. Urdu	12K comment	Abusive/Non-abusive	CNN	96.2% (Urdu), 91.4% (R. Urdu)	DL outperforms ML	Freestyle writing
22	2025	Urdu OLID (SVM/LR)	UOLD (12K tweets)	Offensive/Non-offensive	LR/SVM	86% Acc, 75% F1	Unbiased sampling	Binary-only
23	2025	Sarcasm Detection	Social media APIs	Sarcastic/Non-sarcastic	Transformer (BERT)	Improved results	Multilingual support	Resource-intensive
24	2025	Insult Detection (AdaBoost)	46K comment	Insulting/Non-insulting	AdaBoost	97.79% F1	Cultural nuance preserved	No transformers
25	2025	CMSA-mBERT	CMDSA-24 (48K)	Sentiment (Pos/Neg/Neut)	CMSA-mBERT	79.94% Acc	Code-mixed support	Ambiguous contexts

## 3. Methodology

**Fig 1:**
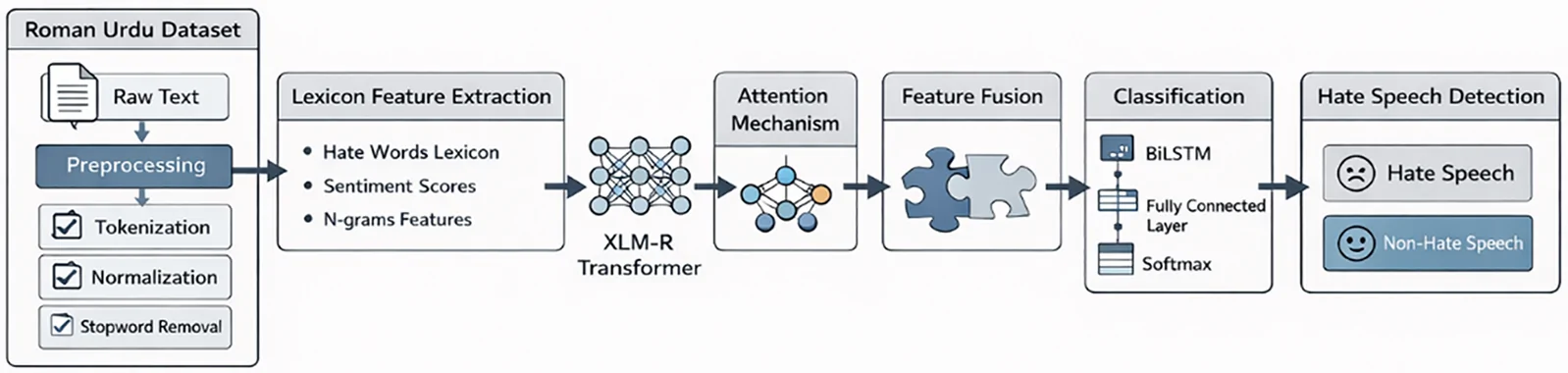
Enhanced LEXF-ATT-XLM Model Pipeline for Roman Urdu hate speech detection.

In this section, the methodology of the proposed Enhanced LEXF-ATT-XLM model for Roman Urdu hate speech detection is discussed. As can be noted, this model combines contextual language modeling and lexicon-based supervision, which helps to enhance performance. The methodology workflow is presented in Fig 1, which shows the workflow from preprocessing to classification and lexicon regression score.

### 3.1. Dataset description and statistics

The category-wise distribution is presented in Table 2. This study operates the RU-HSD-30K dataset, consisting of 30,000 Roman Urdu tweets annotated into Hate (H) and Normal (N) categories [2]. The category-wise distribution is presented in Table 2.

**Table 2:** Category-wise distribution of instances in the RU-HSD-30K dataset.

Category	Number of Instances
Hate (H)	14,801
Normal (N)	15,199
Total	30,000

Although the dataset is relatively balanced, a slight class skew is present. To ensure fair and reliable performance evaluation, weighted evaluation metrics are adopted throughout the experiments.

To obtain robust performance estimates and reduce variance due to data partitioning, we employ 3-fold stratified cross-validation over the entire dataset. Stratification preserves the original class distribution within each fold, ensuring consistent representation of both Hate and Normal classes across training and validation splits. In this setup, each instance is used once for validation and twice for training.

For each fold:

- Two folds are used for training.
- One fold is used for validation.

The process is repeated three times.

Final performance is reported as the average across all folds.

To mitigate optimization bias arising from class imbalance, imbalance handling strategies are applied only to the training partition of each fold. Specifically:

- Minority class samples are upsampled using random resampling with replacement.
- A class-weighted cross-entropy loss is employed.
- A weighted random sampler is used during mini-batch construction.
- Importantly, the validation partition remains unaltered to ensure unbiased and realistic performance evaluation.

### 3.2. Input representation and preprocessing

The model operates on Roman Urdu tweets that undergo specialized preprocessing to handle the linguistic idiosyncrasies of Roman Urdu text. Each preprocessed tweet is tokenized and fed into the XLM-RoBERTa transformer to obtain rich contextual embeddings. To reduce the noise like informal grammar, inconsistent spellings, and code-mixed elements and to preserve semantic cues, a specialized preprocessing pipeline is applied:

- **Character Normalization:** Repeated characters are compressed to a single or acceptable repeat (e.g., “bohottt” → “bohot”) to standardize expressive variations.
- **Negation Preservation:** Negation words (e.g., “nahi”, “mat”) are prefixed to subsequent tokens using a not_ marker (e.g., “nahi samjha” → “not_samjha”) to preserve polarity and syntactic dependencies.

To explicitly preserve negation semantics within contextual embeddings, tokens falling under the scope of a negation cue are transformed using the not_ prefix. This allows the transformer encoder to treat negated and non-negated forms as distinct lexical units (e.g., *samjha* vs. *not_samjha*), thereby reducing polarity ambiguity during representation learning. The transformation is applied only for contextual embedding purposes and does not eliminate the original lexical identity required for downstream lexicon-based processing.

- Removes special characters while preserving alphanumeric and whitespace
- All text is converted to lowercase to unify case-based variations.
- Tweets with missing or null content are excluded. Class labels are mapped to binary form for supervised classification: Hate = 1, Non-Hate = 0.

Each cleaned tweet is then passed to two parallel modules: a transformer-based encoder for contextual embedding and a lexicon scoring mechanism for hate intensity calculation.

### 3.3. Lexicon construction and scoring

Lexicon-guided scoring refers to using a predefined dictionary of offensive words (lexicon) where each word is assigned an intensity score. The model uses this lexicon to enhance its understanding of hate speech by considering these predefined scores. Each word in the lexicon was assigned a weight based on the severity of the word, which ranged from 0.75 to 2.475. These weights are calculated during the lexicon development process.

For a tweet, the lexicon-based hate intensity score is calculated as the sum of weights of all matched offensive terms present in the tweet. Thus, the final tweet-level score can exceed the maximum weight assigned to a term, which is 2.475, in cases where there are multiple hate expressions present in the same tweet. This calculates the additive hate intensity present in the tweet, which is used as an auxiliary regression target but not concatenated with the contextual embeddings.

#### 3.3.1. Lexicon score annotation protocol.

A hate lexicon for Roman Urdu has been created using the training corpus RU-HSD-30K. Frequently occurring abusive words and hate expressions have been identified using frequency filtering. The hate lexicon comprises 1,248 unique hate words.

To capture varying levels of severity, each lexicon term was assigned a graded intensity score in the range:


$$\begin{array}{c} s_{i,k}\in [\text{0}.\text{7}\text{5},\text{2}.\text{5}\text{0}] \end{array}$$

The scale was defined as:

- **0.75–1.00:** Mildly offensive expressions
- **1.01–1.75:** Moderately abusive or targeted insults
- **1.76–2.50:** Highly offensive or explicit hate speech

**3.3.1.1. Annotation procedure:** Three native Urdu speakers with expertise in linguistics and computational text analysis independently rated each lexicon term based on semantic severity and contextual usage in Roman Urdu social media discourse.

**3.3.1.2. Final score computation:** For a lexicon term $t_{i}$ annotated by K=3 annotators, the final intensity score is computed as:


$$\begin{array}{c} S_{i}=\frac{\text{1}}{K}\sum_{k=\text{1}}^{K}s_{i,k} \end{array}$$

where $s_{i,k}$ denotes the score assigned by annotator k.

Final values reflect consensus-adjusted averaging after reconciliation of disagreements.

**3.3.1.3. Inter-annotator agreement:** Annotation reliability was measured using **Krippendorff’s Alpha (α)** for ordinal data:


$$\begin{array}{c} \alpha =\text{1}-\frac{D_{o}}{D_{e}} \end{array}$$

where $D_{o}$ and $D_{e}$ represent observed and expected disagreement, respectively.

For ordinal scoring, disagreement is computed using squared distance:


$$\begin{array}{c} \delta^{\text{2}}(c,c^{\prime })=(c-c^{\prime }{)}^{\text{2}} \end{array}$$

The obtained agreement score:


$$\begin{array}{c} \alpha =\text{0}.\text{8}\text{1} \end{array}$$

indicates substantial inter-annotator reliability.

**3.3.1.4. Lexicon feature integration:** Given an input sequence $X=\{w_{\text{1}},w_{\text{2}},...,w_{n}\}$, the lexicon feature is computed as:

$L(X)=\sum_{w_{j}\in X\cap L}S_{j}$ where L denotes the lexicon set and $S_{j}$ is the intensity score of a matched term. The aggregated score L(X) is incorporated as an auxiliary regression supervision signal within the LEXF-ATT-XLM model to guide hate-sensitive representation learning.

The lexicon score in our model is employed solely as an auxiliary regression signal to provide weak supervision for hate-sensitive representation learning, rather than as a standalone intensity metric. While the raw summation of term intensities reflects cumulative hate cues, explicit normalization (e.g., dividing by tweet length) is unnecessary because the XLM-RoBERTa embeddings and multi-head attention mechanism inherently capture positional and semantic dependencies, enabling the model to distinguish between sparse and dense occurrences. Moreover, the final hate classification is calculated by non-linear transformation and weights, which regulate the saturation effect and prevent the linear scaling of the calculated hate intensity with the number of lexicon hits. The proposed model ensures the effectiveness of the lexicon aggregation for representation learning, while the contextual and non-linear models handle the positional, dilution, and saturation effects.

#### 3.3.2. Consistency between negation preprocessing and Lexicon lookup.

In Section 3.2, during preprocessing, negations are processed. A “not_” prefix is appended to words within the negated scope. For example, “samjha” becomes “not_samjha.” However, during lexicon matching, the original word is used before the prefix is appended.

Specifically, during lexicon feature computation, tokens containing the not_ prefix are decomposed as follows:


$$\begin{array}{c} w_{j}=\left\{ \begin{array}{cc} \text{strip}(w_{j},\text{not}\_) & \text{if prefixed}\\ w_{j} & \text{otherwise} \end{array}\right. \end{array}$$

If the stripped base token exists in the lexicon L, its corresponding intensity score $S_{j}$ is retrieved. However, since the token originated from a negated context, its contribution is attenuated.

Formally, the adjusted score is computed as:


$$\begin{array}{c} S_{j}^{\prime }=\left\{ \begin{array}{cc} \lambda S_{j} & \text{if token contains not}\_\text{ prefix}\\ S_{j} & \text{otherwise} \end{array}\right. \end{array}$$

where λ=0.5 in our implementation.

The final lexicon feature is:


$$\begin{array}{c} L(X)=\sum_{w_{j}\in X\cap L}S_{j}^{\prime } \end{array}$$

This design allows contextual embeddings to retain negation-aware semantics (via the not_ token transformation), while lexicon scoring operates on the canonical base form with controlled attenuation.

### 3.4. Proposed model architecture

**Fig 2:**
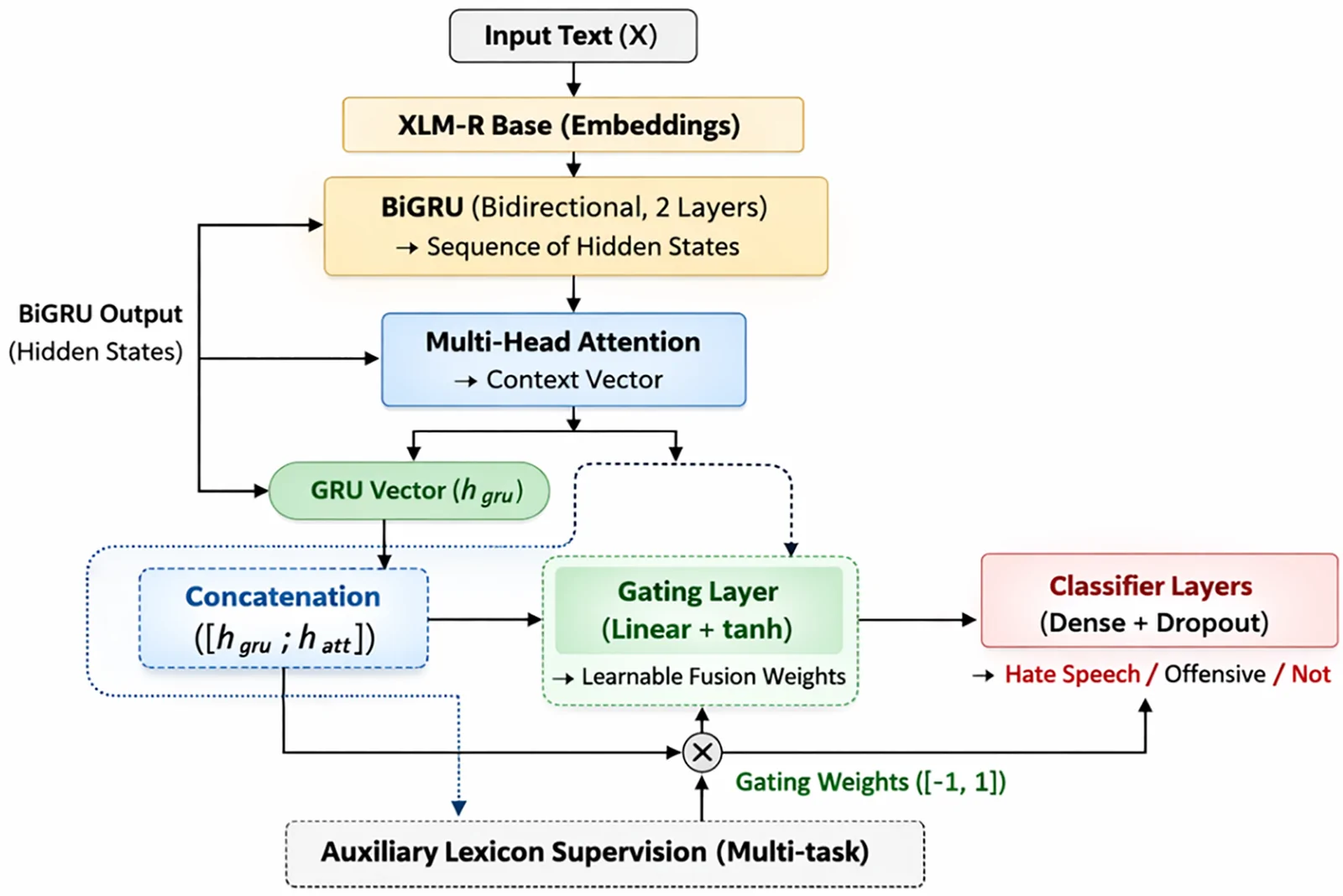
Architecture of the LEXF-ATT-XLM model showing the integration of contextual embeddings, BiGRU, multi-head attention, and lexicon-based auxiliary supervision.

The detailed model architecture integrates contextual language representations and lexicon-guided scoring within a unified deep learning model. As illustrated in Fig 2 the model comprises the following core components:

#### 3.4.1. Transformer-based contextual embeddings.

At the core of this model is the transformer-based model called XLM-RoBERTa. The model generates contextual embeddings for each token. The model has been trained on a large multilingual corpus. As a result, it effectively captures both syntactic and semantic features of Roman Urdu. The tokenized input sequence with a maximum of 256 tokens is passed to the transformer model.

#### 3.4.2. Sequential modeling with Bigru layers.

As the sequential relationships and the word-order-sensitive patterns that use Roman Urdu hate speech, the XLM-RoBERTa token embeddings are taken through a two-layer Bidirectional Gated Recurrent Unit (BiGRU) network. The BiGRU layers work on both forward and backward contexts of a word to enable the net to know how prior words have an effect and subsequent words have on the meaning of a modal word. This layer by layer modeling is very important to identify the minute linguistic elements like negation (“not_bewakoof”) or intensification which normally alters the sentiment and the classification result.

#### 3.4.3. Multi-head attention mechanism.

After the BiGRU layers, the mechanism of a multi-head attention is used to focus on the most pertinent tokens of the input even more. Dynamic attention weights allow each token to be given scores of importance and therefore the network can weight specific words or phrases with great importance particularly offensive or hateful words that are determined by the lexicon. While this provides a form of interpretability, we do not present explicit attention visualizations in this study, and a detailed empirical analysis of attention weights is left for future work.

#### 3.4.4. Auxiliary Lexicon loss for regularization.

Auxiliary regression loss is an additional loss function used alongside the primary classification loss. It enables the model to predict lexicon-based hate intensity scores, reinforcing the classification task by encouraging the model to align its predictions with known hate speech patterns, as represented in the lexicon.

To further guide the learning process, the model incorporates an auxiliary regression loss that predicts the lexicon-based hate intensity score alongside the primary hate/non-hate classification loss. This auxiliary loss acts as a regularizer, encouraging the network to maintain alignment with known lexical hate signals during training. By jointly optimizing classification and lexicon regression objectives, the model reduces overfitting and improves generalization, particularly beneficial in low-resource settings like Roman Urdu.

The fused feature vector is fed to: 1) A binary classifier (2 classes: Hate, Non-Hate), 2) an auxiliary lexicon score predictor (regression head) enforcing alignment between predicted and actual lexicon scores during training.

The pooled BiGRU representation and the pooled multi-head attention context vector are first concatenated and then passed through a learnable fusion (gating) layer implemented as a linear projection followed by a tanh activation. The resulting fused representation is regularized (LayerNorm + dropout) and used as the shared input to (i) the hate/non-hate classification head and (ii) the auxiliary lexicon-score regression head. Lexicon scores are used only as an auxiliary regression supervision signal during training and are not concatenated into the deep feature representation.

#### 3.4.5. Training and optimization.

To tackle class imbalance, both Weighted Random Sampling and a weighted cross-entropy loss were employed. Weighted Random Sampling ensures that there is a balanced batch of hate and non-hate samples during training. At the same time, the weighted cross-entropy loss function assigns greater weights to minority class samples, thus focusing on the correct classification of minority class data. Thus, the overall effect of these two techniques is stability during training, as there is balanced data in batches and at the same time, there is correction at the loss function level.

For the fine-tuning of the transformer-based XLM-RoBERTa model with stability and without overfitting on the relatively small RU-HSD-30K dataset, the learning rate for the AdamW optimizer was set to 1 × 10^(−5), and the model was trained for 4 epochs. Preliminary experiments have shown that increasing the learning rate and the number of training epochs results in overfitting, which is indicated by the deteriorated performance on the validation set. The model has not been subjected to early stopping; instead, 3-fold stratified cross-validation has been used for the model to generalize and prevent overfitting. The cross-validation strategy ensures that the model learns without overfitting the training set due to the gradient steps from the optimizer.

For the prediction of the lexicon score, the cross-entropy loss and the mean squared error loss are used together. The final loss is calculated as a weighted sum of the classification loss and the auxiliary loss:


$$\begin{array}{c} L_{\text{total}}=L_{\text{CE}}+\alpha \times L_{\text{MSE}} \end{array}$$

where $L_{\text{CE}}$ represents the binary classification loss and $L_{\text{MSE}}$ penalizes deviations between predicted and actual lexicon scores. The weight α=0.1 was determined empirically through preliminary validation experiments on a held-out subset of the training data, where values in the range [0.05, 0.2] were evaluated. The best balance between primary classification performance and alignment of the lexicon scores was achieved with a weight of 0.1, so that the auxiliary loss does not dominate the primary objective function while at the same time functioning as a regularizer. This prevents overfitting to the lexicon intensities while still utilizing the hate-sensitive representation learning.

The optimizer employed is AdamW with a learning rate of $\text{1}\times {\text{10}}^{-\text{5}}$, weight decay, and a ReduceLROnPlateau scheduler. Gradient clipping with a max norm of 1.0 and dropout of 0.3 are incorporated for regularization. The model is trained using 3-fold stratified cross-validation to ensure generalization and mitigate overfitting. The training hyperparameters are summarized in Table 3.

**Table 3:** Enhanced LEXF-ATT-XLM model Hyperparameters.

Parameter	Value
Maximum Sequence Length	256
Batch Size	32
Learning Rate	1e-5
Epochs	4
Dropout Rate	0.3
Optimizer	AdamW
Learning Rate Scheduler	ReduceLROnPlateau
Cross-Validation	3-Fold Stratified

The final fused feature vector, which incorporates contextual embeddings from the XLM-RoBERTa model that has gone through the BiGRU and multi-head attention mechanisms, as well as the auxiliary lexicon information, is used to train: (1) a binary classifier that differentiates Hate and Non-Hate text, and (2) a regression model that predicts the lexicon scores. This fusion effectively exploits the contextual and lexicon information together, with the auxiliary lexicon loss aiding in the attention to hate-sensitive tokens while maintaining the contextual information in the transformer model.

The learning rate of 1e-5 was selected after testing several values in preliminary experiments. This learning rate provided the best balance between convergence speed and performance, which is crucial for fine-tuning large models like XLM-RoBERTa. A dropout rate of 0.3 was chosen to prevent overfitting, especially given the relatively small dataset size. Higher dropout rates resulted in underfitting, while lower rates led to overfitting. This dropout rate offered a suitable balance for model generalization

### 3.5. Baseline and ablation study design

To rigorously evaluate and validate the effectiveness of the proposed Enhanced LEXF-ATT-XLM model and empirically verify the contribution of each component of the architecture, we will perform a set of comparisons with basic yet relevant baselines and ablations.

The evaluated configurations include:

- **XLM-R Base:** Fine-tuned XLM-RoBERTa with only a classification head, without the lexicon auxiliary task, BiGRU layers, or multi-head attention. This model serves as the primary baseline.
- **BiGRU:** Removes the sequential BiGRU layers, connecting XLM-R directly to the attention mechanism.
- **ATT:** Removes the multi-head attention mechanism, replacing it with mean pooling.
- **LEXF:** Removes the lexicon scoring auxiliary task and lexicon-based features.

These ablation variants are designed for testing the impact of sequential modeling, attention, and lexicon supervision separately. For testing these ablation models, all of them are trained under similar conditions and compared fairly.

The weighted F1-score is utilized as a primary evaluation metric for these experiments because of class imbalance in the RU-HSD-30K data set. Quantitative evaluation of these ablation experiments is presented in Section 4.

### 3.6. Evaluation metrics

The performance was measured with the help of standard metrics, Precision, Recall, F1-score, and Accuracy which was calculated over all folds. These metrics can provide a comprehensive understanding of the accuracy of the model in terms of the classification results, especially in dealing with the imbalance of the Roman Urdu hate speech data. Measures taken on population level, average metrics over folds were reported, such as Accuracy, Macro F1, Weighted F1, along with class-wise F1-scores, both of Hate and Non-Hate classes and revealed the overall effectiveness of the proposed model and its robustness.

## 4. Results and discussion

This section presents the experimental results evaluating the effectiveness of the proposed Enhanced LEXF-ATT-XLM model for Roman Urdu hate speech detection. We perform our experiments in the Google Colab environment that helped us effectively train and test our suggested model on the RU-HSD-30K dataset using 3-fold cross-validation. The presented model takes the multilingual transformer embeddings (XLM-RoBERTa), lexicon-based features, a BiGRU encoder with attention, and one additional auxiliary lexicon regression head to significantly classify Roman Urdu hate speech. The dataset used contains an almost balanced distribution of hate and non-hate speech instances, as shown in Table 2.

### 4.1. Cross-validation performance

We evaluated the proposed LEXF-ATT-XLM model using 3-fold stratified cross-validation to ensure robustness and generalization across different data splits. For each fold, it was trained for four epochs under identical hyperparameter settings. To measure its performance, accuracy, precision, recall, macro F1-score, weighted F1-score, and class-wise F1-score were considered for Hate and Non-Hate categories. The best epoch for each fold is shown in Table 4.

**Table 4:** 3-fold cross-validation results (Accuracy and F1 scores) on RU-HSD-30K.

Metric	Fold 1	Fold 2	Fold 3	Average
Accuracy	0.8858	0.8919	0.8873	0.8883
Weighted F1	0.8858	0.8919	0.8871	0.8883
Hate Class F1	0.8848	0.8921	0.8912	0.8894
Non-Hate Class F1	0.8867	0.8916	0.8830	0.8871
Macro F1	0.8858	0.8919	0.8858	0.8878

The model attained an average weighted F1-score of 0.8883, indicating balanced performance across both hate and non-hate categories. The model attained an average weighted F1-score of 0.8883, indicating consistent performance across both hate and non-hate categories. However, the RU-HSD-30K dataset is nearly balanced, there is very little difference between the Hate (0.8894) and Non-Hate (0.8871) class F1-score, and it is statistically insignificant. This is a clear indication that the proposed model is able to generalize very well without any bias towards any label. However, it is worth noting that the major challenge with hate speech detection for Roman Urdu is not class imbalance.

Throughout the training process, we observed a consistent reduction in training loss across epochs, with the best performance typically achieved at epoch 4. This convergence pattern highlights the stability of our model architecture in learning from both contextual and lexicon-based cues.

### 4.2. Contributions of methodological components

Each of these components of the model architecture uniquely contributed to the final performance. We used XLM RoBERTa embeddings, which provided us with contextualized representations, especially helpful in handling noisy Roman Urdu text. We used the BiGRU encoder to model the sequential dependencies in the text, while the multi-head attention helped in focusing on the hate-indicative tokens in the text. We used the scores obtained using the lexicon as an auxiliary regression objective, thus emphasizing the hate patterns in the text.

Compared to simpler pooling-based approaches, BiGRU was useful in modeling user text as it can capture sequential dependencies. This was useful in capturing hate/toxic meanings, which are often spread throughout the sentence. Attention was also very useful in the final performance. It helped in focusing on the hate-indicative parts of the text. It also helped in making the model more interpretable since we can trace the decision-making process of the model. It is worth noting that we used the lexicon features as an additional objective. Lexicon scores were incorporated as an auxiliary regression target, providing an additional supervision signal without direct feature-level fusion. This dual usage of lexicon information reinforced hate-related patterns during training and helped align internal representations with the semantic notion of toxicity.

### 4.3. Lexicon-aided predictions and case analysis

To further understand the model’s behavior, we have also carried out qualitative analysis on a wide range of real Urdu text data. Table 5. presents sample predictions along with model confidence scores and lexicon hate scores.

**Table 5:** Sample predictions with model output, probabilities, lexicon scores, and hate term analysis.

Original Text	True Label	Prediction	Probability (Hate/ Non-Hate)	Lexicon Score	Analysis/ Reason for Prediction
Tum jaise chirkut harami kutte ki aulaad ho	Hate	Explicit Hate Speech	0.9999/ 0.0001	3.75	Strong hate terms detected; model and lexicon agree.
Aap ka presentation bohat acha tha	Not Hate	Not Hate Speech	0.0064/ 0.9936	0.00	No strong hate terms; correctly classified.
Main tumse nafrat karta hoon	Hate	Explicit Hate Speech	0.9887/ 0.0113	0.00	Lexicon missed “nafrat”, model relied on context; borderline lexical support.
Yeh cricket match bohat exciting tha	Not Hate	Not Hate Speech	0.0256/ 0.9744	0.00	No hate terms; correctly classified.
Aap ki mehnat qabil-e-tareef hai	Not Hate	Not Hate Speech	0.0365/ 0.9635	0.00	Correctly predicted; neutral content.
Tum logon ko koi sharam nahi hai? Besharam qaum	Hate	Explicit Hate Speech	0.9998/ 0.0002	1.80	Moderate lexicon support; borderline case due to complex phrasing.
Allah tum sab ko hidayat de	Not Hate	Not Hate Speech	0.0177/ 0.9823	0.00	Correct; borderline due to religious context without hate terms.
Tumhare jaise logon se nafrat hai	Hate	Not Hate Speech	0.4300/ 0.5700	0.50	Misclassified: subtle lexical cues, polite phrasing misled the model.
Ye banda pagal hai, par dosti hai	Not Hate	Explicit Hate Speech	0.6100/ 0.3900	0.25	Misclassified: “pagal” flagged as offensive, context shows friendly tone.

Several important insights emerge from these examples. First, the model successfully flags explicit hate speech that aligns with lexicon cues (e.g., high lexicon scores). However, it also identifies hate speech in semantically hateful sentences with zero lexicon score (e.g., “main tumse nafrat karta hoon”), demonstrating its contextual generalization ability beyond keyword matching. This is a direct result of combining deep contextual modeling with lexicon-informed supervision.

On the other hand, a few borderline or sarcastic expressions like “Tumhare jaise logon se nafrat hai” and “Ye banda pagal hai, par dosti hai”, were misclassified or predicted as non-hate, reflecting the current model’s focus on overt hate and highlighting challenges in detecting implicit hate and nuanced intent.

### 4.4. Ablation study results

To empirically verify the contribution of each architectural component in the proposed LEXF-ATT-XLM model, we carried out systematic ablation tests on all variants of the model. These variants include XLM-R Base (a fine-tuned version of XLM-RoBERTa with a classifier on top), BiGRU (no sequential BiGRU layers), ATT (no multi-head attention mechanism, using mean pooling as alternative), and LEXF (no lexicon auxiliary supervision/lexicon features). All models were trained under the same conditions (i.e., using the same data splits, hyperparameters, and evaluation protocol). We use the averaged performance across 3-fold stratified cross-validation with weighted F1 as primary metric. Table 6 presents the ablation study results, demonstrating the weighted F1-scores achieved by the individual components of the proposed LEXF-ATT-XLM model.

**Table 6:** Ablation study showing weighted F1 for components of LEXF-ATT-XLM.

Model Configuration	Weighted F1	Δ from XLM-R Base	Δ from Full Model
XLM-R Base	0.8463	—	−4.73%
BiGRU	0.8703	+2.84%	−2.03%
ATT	0.8733	+3.19%	−1.69%
LEXF	0.8603	+1.65%	−3.15%
Full Model (LEXF-ATT-XLM)	**0.8883**	**+4.96%**	—

The XLM-R base model with fine-tuning yields a weighted F1 of 0.8463, which forms a solid transformer base. Adding BiGRU can enhance performance to 0.8703 (+2.84%), which shows the effectiveness of sequential modeling in modeling phrase-level and contextual interaction in Roman Urdu. Multi-head attention also enhances F1 to 0.8733 (+3.19) meaning that attention assists in refining on salient hate-indicative token. The removal of lexicon supervision (−LEXF) results in a 0.8603, a reduction of 3.15 percent of the full model, which highlights the relevance of explicit lexicon knowledge in the task of informal spelling and code-mixing typical of Roman Urdu. The complete model has a weighted F1 of 0.8883 that is a 4.96 percent increase over the XLM-R baseline. These findings prove that every added element makes a positive contribution to performance instead of merely making the situation more complex.

**Fig 3:**
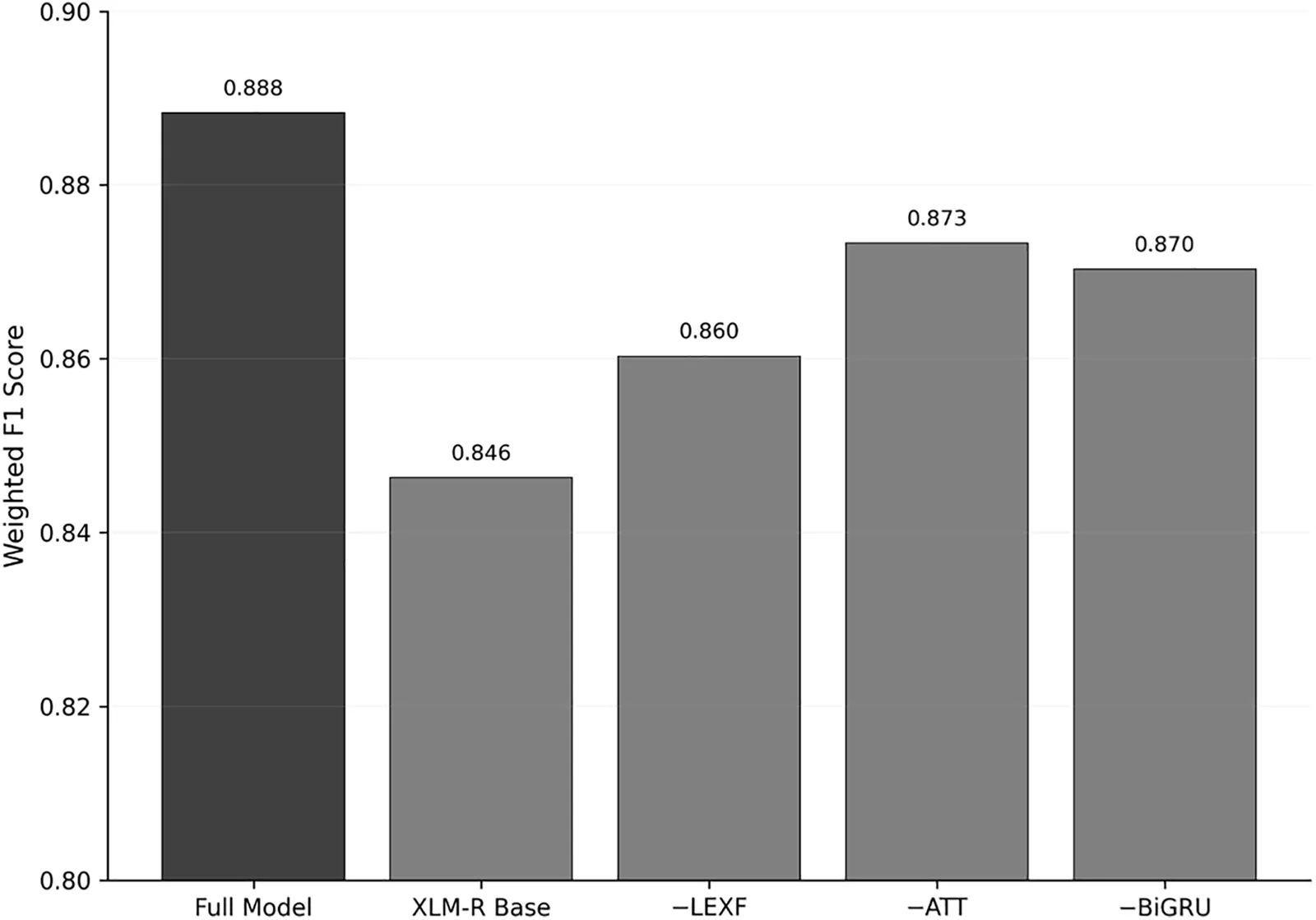
Ablation study showing the effect of model components on weighted F1 (3-fold average) for RU-HSD-30K.

Fig 3 visually demonstrates the changes added by each component which have been incremental. The reliable increasing pattern in the XLM-R baseline to the entire LEXF-ATT-XLM model indicates the cumulative efficiency of sequential modeling, attention processes, and lexicon directed supervision.

### 4.5. Component analysis and trade-offs

The ablation results reveal three key insights:

1. **Lexicon contributions are substantial.** The largest single component drop is seen when lexicon supervision is removed, at −3.15% compared to the full model, which again underscores the benefit of using domain information in low-resource and noisy Roman Urdu.
2. **Attention and sequential modeling are complementary.** Attention (+3.19% over baseline) slightly outperforms BiGRU (+2.84% over baseline), suggesting that focusing on salient tokens is marginally more beneficial than adding sequential capacity alone. Both, however, provide meaningful gains.
3. **Synergy and overlap.** The sum of single-component gains (2.84% + 3.19% + 1.65% = 7.68%) is larger than the observed combined improvement (4.96%), indicating partial overlap in the information captured by different components. Nonetheless, the full model outperforms all partial variants, confirming that the components capture complementary information useful for detection.

Practical implication: For low-resource languages such as Roman Urdu, integration of lexicons is of utmost importance, followed by attention and sequential modeling as subsequent improvements.

Limitations. We report average cross-validation metrics. However, statistical testing (e.g., paired t-tests over folds, or confidence intervals using bootstrapping) of metrics for all pairwise comparisons is not carried out here and is left for subsequent work.

### 4.6. Comparison with state-of-the-art

We compare LEXF-ATT-XLM to prior representative work on Roman Urdu hate speech detection, evaluated on the same dataset RU-HSD-30K for fair evaluation. Table 7 presents a comparative evaluation of the proposed LEXF-ATT-XLM model against existing state-of-the-art approaches on the RU-HSD-30K dataset.

**Table 7:** Comparison of LEXF-ATT-XLM with prior work on RU-HSD-30K.

Study	Model Architecture	Dataset	Accuracy	F1-Score
Jan et al. [2]	Bi-LSTM + Attention + Word2Vec (custom)	RU-HSD-30K	0.875	0.885
Our Work	**LEXF-ATT-XLM**	**RU-HSD-30K**	**0.8883**	**0.8883**

On RU-HSD-30K, our model improves over Jan et al. [2] by +0.33% F1 (0.8883 vs. 0.885). Although the differences in the datasets, annotation schemes, and evaluation protocols make it difficult to draw any hard and fast comparisons, the overall trend in performance improvements in all ablations and baseline comparisons suggests that lexicon-based multi-task learning, along with the sequential and attention modules, is useful in Roman Urdu hate speech detection.

### 4.7. Overall observations and future directions

The effectiveness of our hybrid model is justified by the fact that it has a strong overall performance and, in particular, has the capability of generalizing and is not limited to simple key word spotting. The interpretation of lexicon scores offered using either concatenation or regression forms offered interpretability and regularization to the model to make the distinction between high-intensity hate, mild-toxic language, and benign language.

**Fig 4:**
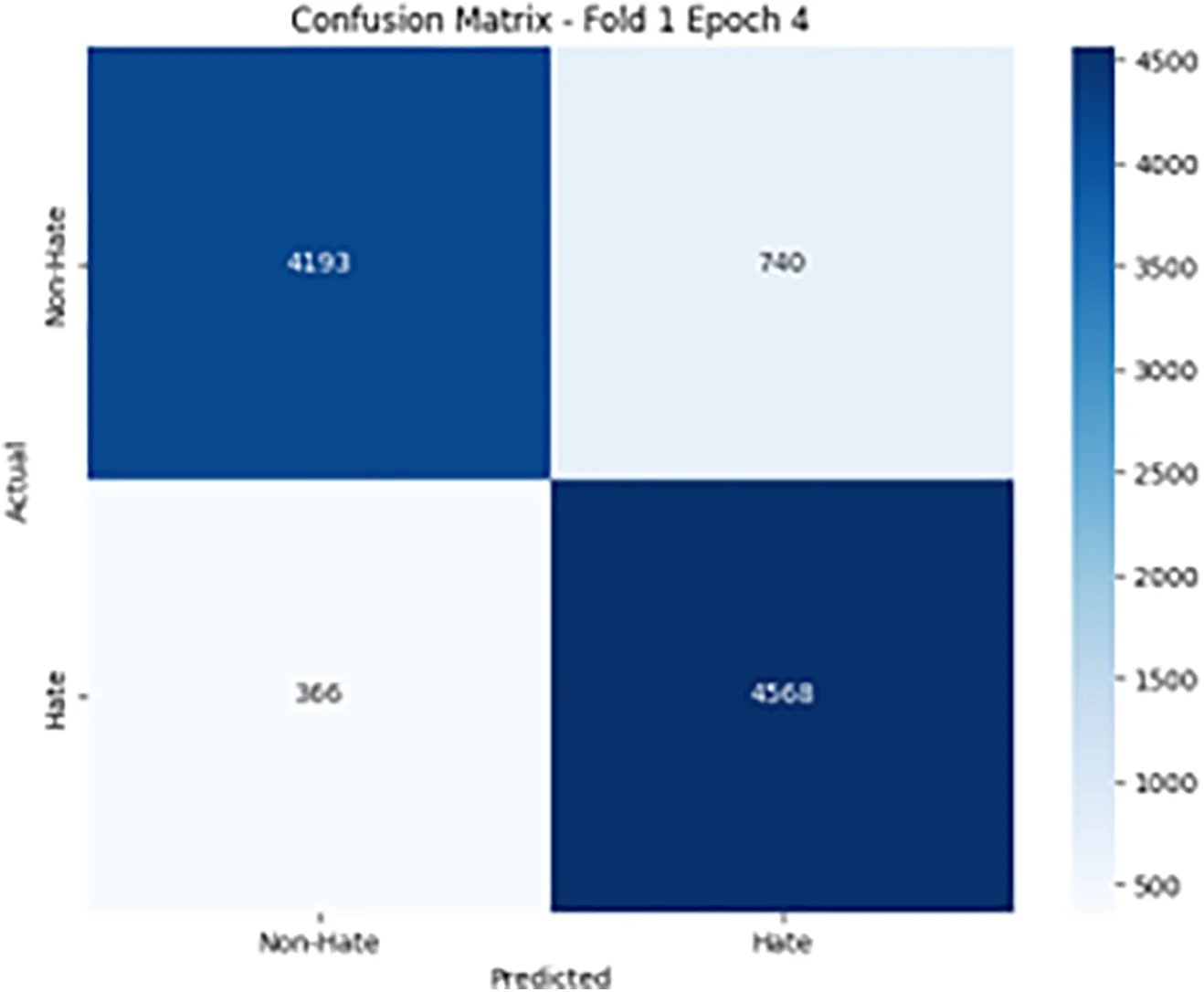
Confusion matrix for LEXF-ATT-XLM predictions on RU-HSD-30K.

Fig 4. Summarizes the model’s performance at Fold 1, Epoch 4. The LEXF-ATT-XLM model accurately classified most hate and non-hate instances, with relatively few misclassifications. It showed strong sensitivity to hate content, reflected by a low number of false negatives, and maintained solid performance on non-hate data despite slightly higher false positives. The accuracy, recall, and F1-scores were balanced well in both classes. Additional support of the model is confirmed by the macro and weighted averages to control the imbalance in classes. This was the most successful period of Fold 1, which denotes the stability of the model in the hate speech recognition in Roman Urdu.

The model however, sometimes fails in pragmatic understanding, especially in sarcastic, coded, or indirect hate cases. In future work, this could be addressed by integrating additional pragmatic features, sentiment cues, emoji/token context, or conversation structure. Attention weight visualization and interpretability analysis will also be explored in future work, which can provide deeper insights into the decision-making process of the model. Moreover, training the model on larger or more diverse corpora of Roman Urdu, possibly with the help of data augmentation or pseudo-labeling techniques, can be helpful for better generalization. To conclude, the proposed Enhanced LEXF-ATT-XLM model offers a linguistically well-informed and precise solution for the detection of hate speech in the Roman Urdu language, which can be extended to similar low-resource and code-mixed language scenarios.

## 5. Conclusion

This paper proposed a sophisticated hybrid deep learning model, the LEXF-ATT-XLM, to solve the complex problem of hate speech detection in Roman Urdu. Using a combination of XLM-RoBERTa embeddings, BiGRU sequential modeling, and multi-head attention and a learnable gating mechanism helped us not only to capture the global context but also to capture the local linguistic nuances. The main innovation of the combination of a domain-specific hate lexicon using an auxiliary regression supervision signal allowed the model to use explicit toxicity cues without affecting the integrity of deep contextual features. The proposed model evaluated on the RU-HSD-30K dataset with 3-fold stratified cross-validation had an average accuracy of 88.83% and an average weighted F1-score of 88.83%, and it performed consistently across the folds (88.58%, 89.19 and 88.71). We empirically confirmed our hypothesis that the combination of lexicon-guided auxiliary supervision, sequential modeling and attention mechanisms generate unique, complementary performance advantages by our ablation studies, demonstrating the effectiveness of our method in overcoming orthographic inconsistency, code-mixing and negation ambiguity. Although our framework is highly effective at detecting explicit hate speech, future efforts will be directed towards extending this multi-task framework to multi-class conditions to detect finely-grained classes of hate and the incorporation of multimodal data to further refine decision-making in low-resource conditions.
